# Shape-Based Alignment of the Scanned Objects Concerning Their Asymmetric Aspects

**DOI:** 10.3390/s21041529

**Published:** 2021-02-23

**Authors:** Andrej Lucny, Viliam Dillinger, Gabriela Kacurova, Marek Racev

**Affiliations:** 1 Department of Applied Informatics, Faculty of Mathematics, Physics and Informatics, Comenius University, Mlynska Dolina, 842 48 Bratislava, Slovakia; 2 ME-Inspection SK, Drobneho 25A, 84101 Bratislava, Slovakia; viliam.dillinger@me-inspection.sk (V.D.); gabriela.kacurova@me-inspection.sk (G.K.); marek.racev@me-inspection.sk (M.R.)

**Keywords:** shape-based object recognition and alignment, Retinex filter, Triangle Area Representation, cyclic Dynamic Space Warping, Kabsch algorithm

## Abstract

We introduce an integrated method for processing depth maps measured by a laser profile sensor. It serves for the recognition and alignment of an object given by a single example. Firstly, we look for potential object contours, mainly using the Retinex filter. Then, we select the actual object boundary via shape comparison based on Triangle Area Representation (TAR). We overcome the limitations of the TAR method by extension of its shape descriptor. That is helpful mainly for objects with symmetric shapes but other asymmetric aspects like squares with asymmetric holes. Finally, we use point-to-point pairing, provided by the extended TAR method, to calculate the 3D rigid affine transform that aligns the scanned object to the given example position. For the transform calculation, we design an algorithm that overcomes the Kabsch point-to-point algorithm’s accuracy and accommodates it for a precise contour-to-contour alignment. In this way, we have implemented a pipeline with features convenient for industrial use, namely production inspection.

## 1. Introduction

Laser profile sensors [1,2] provide very accurate depth maps at a very reasonable speed suitable for a wide range of useful tasks. Their typical final output is a 2D array containing distances from the sensor to the scanned area for each point in a 2D grid. Each such sensor measures a limited range; thus, some values may be non-measured. We can view this data as an image in which pixel values are float numbers (z-coordinate) or not-a-numbers. On the other hand, we can display them also as a 3D point-cloud. For any reasonable use, we have to process the data by various methods of computer vision. We introduce one such processing pipeline in this paper.

Object recognition and alignment [3] are tasks frequently solicited in various domains of industry [4]. Having a single template, we have to identify the corresponding object in a scanned depth map and calculate the transform that aligns the found object into a default position, orientation and size given by the template. The recognized object is potentially displaced, rotated, or scaled; thus, we aim to find an accurate rigid affine transform [5]. Then we use the transform for a particular task like quality inspection [6,7,8]. We typically need to align objects like various housings, holders, straps, teeth wheels and others. After alignment, we can check their dimensions, completeness of details (screws, letters), or defects (missing areas, extra areas).

Though we can employ various methods for implementing our processing pipeline, it is challenging to look for a general solution for real industrial needs. The available strategies suppose substantial limitations to the number or shape of the scanned objects [9]. Concerning a general-purpose, no well-known image registration method has been found that is suitable for our purposes. The classic approach of the phase correlation [10] is fast (thanks to the Fast Fourier Transform algorithm) but limited to the same size and rotation, while its improvements [11] are not sufficiently accurate. Methods based on image features (SIFT, SURF, ORB [12]) are not suitable for our purpose at all. Though [13] employs them for shape-based inspection under very similar circumstances, they are not general enough. These methods do not work for fade objects that do not provide enough key-points [14]. Finally, the Efficient Correlation Coefficient (ECC) [15] can gradually find the transform of a template image onto a part of a given image, but for more rotated objects with complicated shapes, it gets stuck in a local minimum and does not reach the optimal alignment. Moreover, using ECC, we have to estimate the initial object position with another method.

During our technology pool, we found the shape-based methods much more applicable to our mission. In [16], the processing pipeline for calculating object position and distance successfully employed the Hough transform [17], applied after the Gaussian filter, edge detection and threshold-based segmentation. In [18], a similar processing pipeline consisted of binarization, centroid location with the linear displacement calculation, edge detection and angular displacement calculation is also shape-based. The pipeline presented in [19] based on the Farthest Point Angle descriptor contains shape representation with contour, signature construction, shape description using Fourier Transform, shape toning and ranking.

The best shape-based method we found is Triangle Area Representation (TAR) [20,21,22]. It is based on the Dynamic Space Warping (DSW) algorithm [23]. TAR provides not only the distance between two contours concerning their similarity but also their point-to-point pairing. Thus, we can directly employ its output as input for their alignment. This method rarely occurs in industrial applications, but some applications have already existed. Reference [24] measured geometrical properties of profiled fibers. Reference [25] implements fiber classification. These applications deal with inspecting a concrete object, while our approach is general—we do not know what particular objects we process.

However, the shape-based methods also have limitations we need to overcome. Frequently, the outer contour of objects is not sufficient for their proper alignment. Even when shapes align correctly, the inner areas can align in the wrong way if we process symmetric objects with some asymmetric aspects. Some specific shape descriptors already designed can solve this problem. The Hybrid Shape Descriptor (HSD) [26,27] is based on circular areas covering the object. The inner part of objects is partially concerned with the shape descriptor presented in [28]. In general, these descriptors are less efficient, and require a perfect image binarization. That makes them not applicable for our purposes. Therefore we aim to use TAR and improve it by a solution to this problem. We follow the idea from [29], which presents an approach to the shape descriptor extension. They extend the shape descriptor by the texture and color, so we cannot employ their approach directly for our depth maps. However, their success indicates that such extensions can solve this problem.

Since TAR does not apply directly to images, we need another method for finding potential object contours. That is also not a trivial task. The most common edge detectors are not suitable here because the edges they provide are not always continuous. We have to binarize the image to ensure the continuity of contours. When we scan objects lying on the floor outside the sensor range, the binarization is straightforward—we distinguish just the measured and non-measured values. However, we need a sophisticated approach when the scanner detects one object lying on another one or the floor. No local thresholding method, like Otsu, MaxEntrophy, Niblack, Sauvola or others [30], is perfect. However, we can successfully use an arbitrary one if we preprocess the image with the Retinex filter [31]. The output from the filter we binarize by the IsoData [32] local thresholding approach. Remarkably, methods that do not require continuity of edges have already existed like Contour Segment Context (CSC) [33]. However, phantom detections’ danger is serious when we are concerned with more kinds of objects that may or may not be present in the scanned data. Having a single edge, we can eliminate phantom detection by its shape. But when we have more ones per object, their mutual placement can generate phantoms.

Finally, having point-to-point pairing, we had to calculate the rigid affine transform that aligns the recognized object contour onto the template object contour. We expected that it is enough to call the Kabsch algorithm [34] for that (e.g., [35] employs it for the same purpose), but we encountered troubles caused by the fact that the contours are sequences of the sampled points. We are not looking for a point-to-point transform but a contour-to-contour one. And unlike typical contour-to-contour alignment algorithms [36], we have to preserve shape. In [37], the proper algorithm is designed for 2D; however, we need 3D.Therefore we had to design our solution, more sophisticated than the Kabsch algorithm. A similar problem with alignment accuracy has appeared in [38]. Our solution employs a similar idea to that in [39].

In this paper, we present our solution for the complete process pipeline to the above problem. Its quality is good enough for use in practice. Our solution stems from the composition of existing methods. Besides their integration, our contribution is their improvement by resolving issues that are beyond their capacity. We present it in the next section. We gradually describe the processing pipeline, starting with contour detection, following the shape comparison and its extension introduced by us, and ending with our alignment algorithm. Further sections present results and discuss their quality and potential.

## 2. Materials and Methods

**Figure 1:**
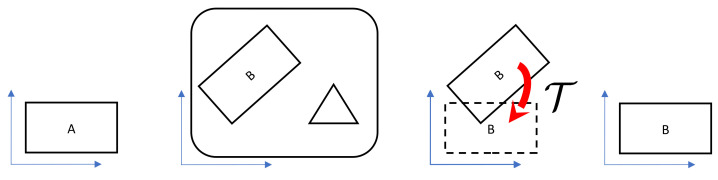
The objects recognition and alignment tasks. From left to right: template, scan, the recognized object with transform, the aligned object (top view of 3D scenes).

We designed a method for the object recognition and alignment task concerning inspection purposes (Figure 1). The process’s input is a depth map provided by the laser profile sensor. Its output is an affine transform that aligns the recognized object to a standard position. We have a database of templates that describes a set of the inspected items, created by manual selection of their contours on scans made when they are in the standard position. During the object identification, we processed the measured data as the 2D array of measured elevations in which some points can be missing. After the object recognition, we considered the data a 3D point-cloud, in which alignment we find (Figure 1).

**Figure 2:**
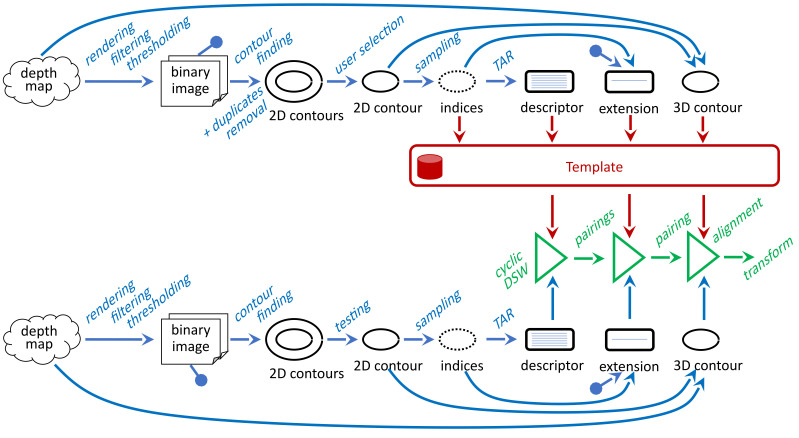
Our processing pipeline for object recognition and alignment.

Our processing pipeline for object recognition and alignment is as follows (Figure 2). In the beginning, we scanned the template object in its standard position and rotation. We process the scanned depth map to a list of present contours and offer them to the user. The processing resides in the point-cloud rendering to the 2D image, its binarization and contour finding. Since we can use more binarization methods, we also have to address the removal of duplicates. The user selects one contour that corresponds to the object boundary. Then, we process the selected part of a template and store it for later use. At first, we sampled its shape by a fixed number of points, so we get indices that we could use to index both the 2D contour and the corresponding points of the 3D point-cloud. Secondly, we calculated the shape descriptor from the indexed 2D points. Thirdly, we described the inner part of the object by the so-called extension. Later we scanned scenes with translated and rotated objects that the template represents. We processed the scanned depth map in an analogical way and compared each found contour with the template. The comparison does not only identify the demanded item, but we also get a pairing of its outline points with the outline points of the template. Then we could use the pairing for the best matching item for the calculation of the alignment transform. If we get more candidates for the best matching, we select one of them, following the extension. Finally, we aligned the scanned depth map by the calculated transform.

The mentioned processing relies on several separated methods applied gradually. At first, we have to find contours in the scanned depth map. Then we have to implement their shape based comparison, which can also provide their pairing. Finally, we have to turn the pairing to the alignment transform. We describe these three steps in the following subsections.

### 2.1. Finding Contours

Contours we are looking for must be closed and continuous. We can grant this feature if we turn the scan into a binary image and process it by the traditional contour finding algorithm [40]. We are concerned with more methods of binarization, so we put together more sets of found results. We rely on two approaches. The main one employs the Retinex filter [31]. The filter turns an image into a logarithm of the ratio between pixel value and its average vicinity value. The average utilizes the Gaussian filter of a bigger size that can be effectively calculated by the circular convolution. That is fast due to the employment of the Fast Fourier Transform algorithm. Thus, the Retinex filter *R* of image *I* is calculated as follows:

(1) $$R\left(I\right)=\log\frac{I}{G*I}=\log I-\log G*I=\log I-\log F^{-1}\left(F\left(G\right)F\left(I\right)\right),$$

where *G* is the 2D Gaussian filter, *, is the convolution operator and F denotes the Fourier Transform.

**Figure 3:**
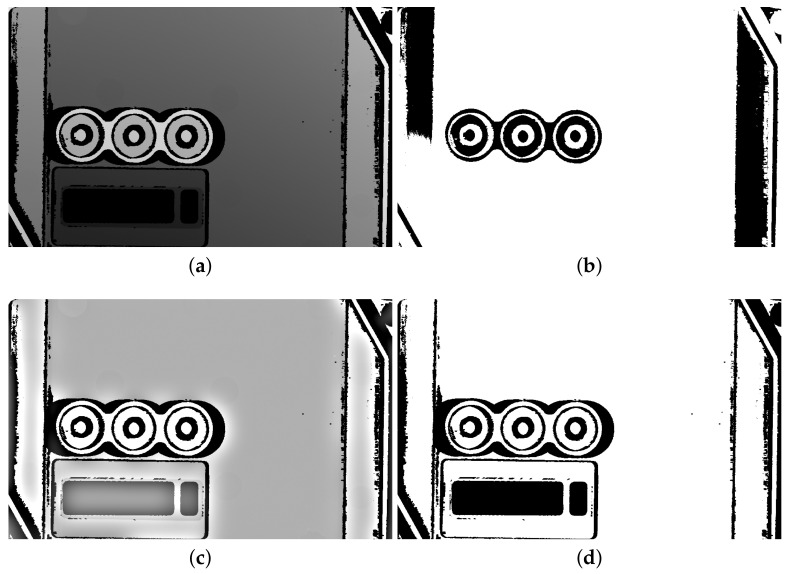
Retinex filter. (**a**) The scanned depth map; (**b**) binarization of the scanned depth map by the IsoData threshold; (**c**) the output from the Retinex filter applied to the scanned depth map; (**d**) binarization of the Retinex filter output by the IsoData threshold.

The Retinex filter does not binarize images but turns the majority of pixel values to very low or very high. Thus, it is easier to binarize its output by a suitable threshold; we employ IsoData local threshold [32] for this job (Figure 3).

Though the Retinex filter nicely preserves contours, it also generates phantom ones at each flat area in which there is another smaller and higher object. Thus, the stepped pyramid has not only square outlines but also volatile ones at each flat step. However, concerning the contours hierarchy, it is easy to ignore them.

There is just one case in which the Retinex filter fails. That is a scan of a single object, which has high and low parts. If there is also a floor, the filter works. However, without the floor, it disintegrates the scanned object. Fortunately, in this case, we can find the proper contour by a straightforward binarization method, which concerns just the measured and non-measured values.

Finally, we join the results of the two methods mentioned above. In the case that we present the found contours to the user for the selection of one corresponding to the represented object, we have to also solve the removal of duplicates. We resolved this by rendering the found contours in downsampled images and comparison of the binary masks. See Algorithm 1.
**Algorithm 1** Contours finding algorithm1:**procedure** FindContours(*I*)                     ▷*I* is a depth map2:  Contours←{}               ▷ list of closed 2D point sequences3:  Add contours from IsoData(Retinex(*I*)) to Contours4:  Add contours from IsMeasured(*I*) to Contours5:  Remove duplicates in Contours                   ▷ optional6:  **return** I(Contours)          ▷ return list of closed 3D points sequences

### 2.2. Shape-Based Comparison

**Figure 4:**
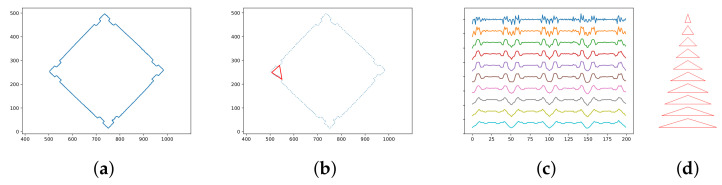
Shape descriptor based on the Triangle Area Representation (TAR). (**a**) Contour; (**b**) sampled contour (200 points) with an example of the considered triangle; (**c**) TAR descriptor (each line represents 200 normalized triangle areas; each area corresponds to point and its close neighbors) (**d**) illustrative triangles (indicating how far the neighbors are from the point).

Our approach to shape comparison is based on TAR [20]. Each contour in a depth map is a sequence of adjacent 2D points. The amount of points varies, but we sample them to a fixed number. It is reasonable to be concerned with *l* points, where *l* equals 100 or 200. More points better describe the shape; fewer points enable fast processing. We process the sampled contour to the so-called TAR descriptor—a set of sequences of the normalized triangle areas. Each one contains areas of triangles in which vertexes are points of the contour. The second and third vertexes are the left and right neighbors of the first vertex. For the first sequence, the distance between the first vertex and its neighbors is one. For the second one, it is two, and so on. The usual number of such sequences *t* is ten. Thus, the TAR descriptor forms a matrix of t×l of values. The triangles’ area can be positive or negative, depending on the angle’s reflexivity at the first vertex. We divide it by the sum of the areas’ absolute values, so it is normalized to the range from −1 to 1. Thus, we have *t* values forming a vector for each point on the sampled contour that describes the local shape. As a result, we can compare the shape at two points by calculating the Euclidean distance between their corresponding vectors. See Figure 4 and Algorithm 2.
**Algorithm 2** Calculation of the TAR descriptor1:**procedure** CalculateTAR(contour)         ▷contour is a sequence of 3D points2:  indices←Sample(contour,l)              ▷ select *l* equidistant points3:  points←contour[indices]4:  Allocate TAR                          ▷ matrix t×l5:  **for** i←1 to *t* **do**6:    **for** k←0 to l−1 **do**7:     $TAR_{i-1,k}\leftarrow$TriangleArea$\left(points_{(k-i)\%l},points_{k},points_{(k+i)\%l}\right)$8:  **return** TAR,indices             ▷ return TAR descriptor and indices

The calculation of TAR descriptors is fast, but their comparison is time consumable; therefore, we have to sample to compare contours. We performed it using the cyclic DSW algorithm [23]. The cyclic DSW is called standard DSW (non-cyclic version) for each possible shift between compared descriptors. We can shift just the second descriptor, so DSW is called *l* times. DSW compares the two descriptors ($r=r_{0},r_{1},\ldots ,r_{l-1}$ and $s=s_{0},s_{1},\ldots ,s_{l-1}$ where $r_{i}$ and $s_{i}$ are vectors of length *t*) and returns minimal distance and point-to-point pairing. If we imagine the best pairing, there is a shift that pairs the first points of *r* with the first point of the shifted *s*. Therefore we can investigate just pairings starting at the beginning of the first descriptor and the shifted second descriptor. The DSW algorithm is a kind of dynamic programming, so it gradually calculates table $D_{i,j}$ containing the minimal distance between the first descriptor from beginning to position *i* and the shifted second descriptor from beginning to position *j*. It employs the following formula:

(2) $$D_{0,0}=\left\|r_{0}-s_{0}\right\|$$

(3) $$D_{i,j}=min\left(D_{i-1,j-1},D_{i-1,j},D_{i,j-1}\right)+\left\|r_{i}-s_{j}\right\|,$$

where the norm is L1.

Since we can suppose that sampling of contours causes just a little variation, it is enough to evaluate *D* values close to diagonal. Therefore, we consider parameter *w*, called a window, which specifies that we examine $D_{i,j}$ for i−w≤j≤i+w and set other values to infinity.

Finally, $D_{l-1,l-1}$ tells us the resulting minimum. Then, we can find a path from (l,l) to (0,0), which corresponds to its gradual calculation. Then, we take each pair from the way to the resulting pairing (in the opposite order). As a result, the pairing does not have exactly *l* pairs, and some points from one contour can pair with more points in the second contour, but in practice, this is still a piece of handy information about their alignment.

We can now evaluate the convergence rate of the DSW algorithm as O(twl); thus, for the cyclic DSW, it is $O(twl^{2})$.

**Figure 5:**
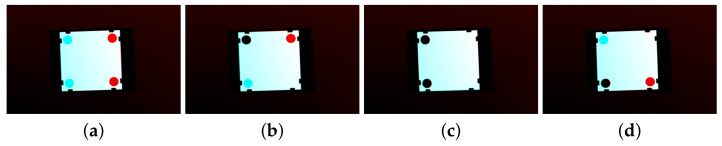
An example of an object with symmetric shape and asymmetric aspects. (**a**–**d**) Aligning by the outer shape, there are four possible alignments, but only one (**c**) is correct. (The template object is cyan, and the aligned one is red. Thus pure cyan represents a missing area and pure red an extra area.)

For most shapes, the integration of results for individual shifts is easy—we select the minimum. However, for symmetric shapes, we get the (almost) same minimum more times. Rectangles and ovals give the minimum two times, squares four times and circles *l* times. If the objects have symmetric not only contour but also the inner area, we can return anyone. However, when their inner area is asymmetric—for example, because of holes—we encounter a limitation of shape-based methods. Imagine, for instance, a square-shaped component with holes in two corners (Figure 5) or a teeth wheel with a hole in one tooth. Such objects provide us more choices on aligning them with their templates; however, only one of them is correct.

**Figure 6:**
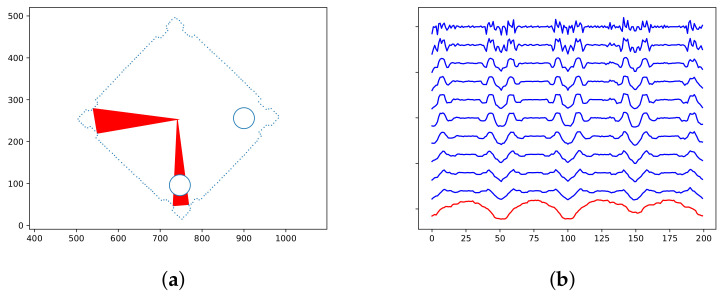
Extension of the TAR descriptor. (**a**) the sampled contour and holes with examples of triangle areas describing the inner object area. (**b**) the TAR descriptor with our extension (the bottom line). It is quite visible that the standard TAR values contain four same segments, whereas the extension two plus two. Thus alignment is uniquely determined.

Fortunately, TAR is suitable for an extension we have invented to solve this problem. We added one additional sequence to the TAR descriptor that describes the inner area of the object. It specifies the normalized area of the triangle with vertexes in left and right neighbors for the last TAR sequence and the center of mass. Of course, we regard only the area covered by the object. We calculated it from the binarization output. Though the Retinex filter is not suitable for this (because of phantom holes inside the object), we can employ the less sophisticated method, which usually cannot find the contour, but sufficiently describes the inner part. In this case, the areas are always positive, so we normalize them into the interval from 0 to 1 (Figure 6). See Algorithm 3.
**Algorithm 3** Calculation of the TAR extension1:**procedure** CalculateExtension(contour,indices,depthMap)2:  mask←isMeasured(depthMap)3:  points←contour[indices]      ▷ the same points as for the TAR descriptor4:  center←Average(points)5:  Allocate Extension                     ▷ vector of *l* values6:  **for** k←0 to l−1 **do**7:    triangle←mask&Triangle$\left(points_{(k-i)\%l},center,points_{(k+i)\%l}\right)$8:    $Extension_{k}\leftarrow$Area(triangle)9:  **return** Extension              ▷ return the TAR descriptor extension

The extension enables us to select the proper minimum and pairing. Of course, we have to assume that the minima can vary a bit. Therefore we examine all minima close enough to the absolute minimum. However, then we would process for each demanded minimum also values returned for similar shifts. We have to use non-maximum suppression for their elimination. In this way, we have extended the TAR method to find the right point-to-point pairing even for objects with symmetric shapes and other asymmetric aspects. See Algorithm 4.
**Algorithm 4** Shape comparison algorithm1:**procedure** Compare(r,s)        ▷*r* and *s* are extended TAR descriptors with length *l*2:  Results←{}3:  **for** k←0 to l−1 **do**4:    Call DSW$(r_{TAR},$shiftBy$\left(s_{TAR},k)\right)$; it returns distance *d* and pairing *P*5:    Add (d,P,k) to Results  6:  $d_{min}\leftarrow \min\left\{d|(d,P,k)\in Results\right\}$          ▷ Find the minimal distance7:  $Minima\leftarrow \{\left(d,P,k\right)|\left(d,P,k\right)\in Result\wedge d\leq d_{min}+\epsilon \}$8:  Minima←NonMaximumSuppression(Minima)        ▷ promising results9:  $best\leftarrow argmin\{\|r_{extension}-s_{extension}\||\left(d,P,k\right)\in Minima\}$10:  (d,P,k)←Minina[best]11:  **return** d,shiftBy(P,−k)      ▷ return the minimal distance and the best pairing

Of course, we can use this mechanism not only for contours comparison but also for object recognition. We process the scan to a set of contours and compare each one with the template. For the demanded object, the mechanism returns a distance close to zero. For others, we get significantly higher values.

### 2.3. Alignment Transform

**Figure 7:**
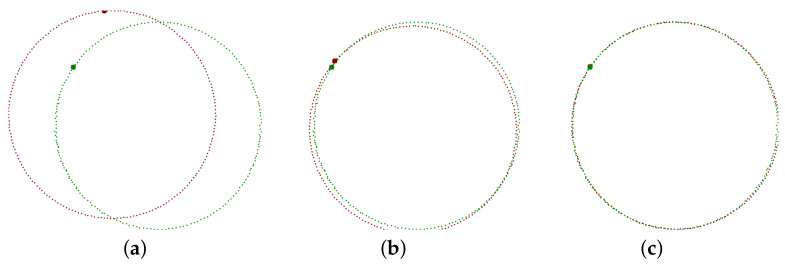
Alignment of 3D contours. (**a**) Inputs to the alignment process; (**b**) the result provided by the Kabsch algorithm; (**c**) the output of our algorithm targeted to contours alignment.

Seemingly, having the best point-to-point pairing, we can get a rigid affine transform that aligns the recognized object straightforwardly. Nothing could be further from the truth. Though we have a standard algorithm that calculates the best transform for a set of pairs of points, its results are insufficient. The problem is that the mentioned Kabsch algorithm [34] is a least square method that minimizes the sum of distances between the transformed origin points and the target points. However, our mission is quite different. Deviations between points lying on the contour are much better than deviations oriented perpendicularly. The contour sampling has just a little impact on the TAR method. However, here it has significant and destructive effects (Figure 7b).

We have found that we can use the Kabsch algorithm as an appraise that we can improve iteratively. The idea is the following. Let the pairing provided by the TAR method contains *n* pairs of points (we notice that *n* can be slightly higher than *l*). We will represent it as three lists of *n* elements—the original points *p*, target points *q*, and indices *k* such that $p_{i}$ pairs with $q_{k_{i}}$. Then we apply the Kabsch algorithm, which gives us a transform T that transforms $p_{i}$ to $T(p_{i})$. Now can investigate what $q_{j}$ is closest to $T(p_{i})$. If the algorithm works for us, $j=k_{i}$. However, there can be a small difference from the beginning, and *j* will differ from $k_{i}$. It will not differ too much; it will be $k_{i}+u$ or $k_{i}-u$ for a little *u* (this feature enables us to find it efficiently). The difference indicates that our pairing is not perfect. So we adjust the pairing by assigning $k_{i}:=j$. And then we repeat the process until we can improve neither index $k_{i}$. See Algorithm 5.
**Algorithm 5** Alignment algorithm1:**procedure** Align(a,b)       ▷*a* and *b* are sequences of 3D points of the same length2:  Start with the initial pairing $a_{i}$ with $b_{i}$ for all *i*3:  **repeat**4:    Call the Kabsch algorithm for the current pairing. It returns a transform T5:    Transfrom points *a* to points c←T(a)6:    Calculate a better pairing: pair $a_{i}$ with $b_{j}$ that is the closest *b* point to $c_{i}$7:  **until** the pairing is not improved8:  **return** T         ▷T is a matrix 3x4 representing a rigid affine transform

Our iterative algorithm is slower than the Kabsch algorithm, which we usually call for real data up to twenty times. However, it provides excellent alignment (Figure 7c). It is not trivial to tell how much it is better. If we have the sum of Euclidean distances of the transformed and target points, it is less (of course) for the Kabsch algorithm because it is optimal for point-to-point matching. But, this is not the measure we want to minimize. It is better to define the contour-to-contour alignment quality by overlapping areas surrounded by the transformed (*A*) and target (*T*) points.

(4) $$Error=1-\frac{\left|A\cap T\right|}{\left|A\right|}.$$

While the Kabsh algorithm error for the sample in Figure 7 is 3.7%, we can decrease it to 0.9% by our method.

### 2.4. Integration

We integrate the approaches mentioned above in a system relatively quickly, but still, we have to finalize some issues. We need to answer the question—what information we have to store as the template representing the demanded objects. Answering it, we can prefer minimal data to be stored or the most efficient calculation. The key reason for following the second approach is that it is good to keep all points of the found contour for its later presentation to the user. These data overwhelm the rest; hence it is reasonable to save all the stuff. Thus, the template contains the following:

- n adjacent points of the found contour, including their pixels’ position and value (we set this data as 3D points).
- *l* indices to the above list that sample the contour for the shape representation purposes
- TAR descriptor t×l values from $\langle -1,1\rangle$ describing the shape
- TAR extension *l* values from $\langle 0,1\rangle$

The overall processing pipeline joins the above-methods. Its summary is in Algorithm 6.
**Algorithm 6** Object recognition and alignment1:**procedure** PrepareTemplate(*A*)                      ▷*A* is a depth map2:  $Contours^{A}\leftarrow$FindContours(A)3:  $Contour^{A}\leftarrow$Select$\left(Contours^{A}\right)$           ▷ Let the user select one contour4:  $Descriptor_{TAR}^{A},Indices^{A}\leftarrow$CalculateTAR$\left(Contour^{A}\right)$5:  $Descriptor_{Extension}^{A}\leftarrow$CalculateExtension$\left(Contour^{A},Indices^{A},A\right)$6:  SaveTemplate$\left(Contours^{A},Indices^{A},Descriptor_{TAR}^{A},Descriptor_{Extension}^{A}\right)$7:**procedure** UseTemplate(*B*)                        ▷*B* is a depth map8:  LoadTemplate$\left(Contours^{A},Indices^{A},Descriptor_{TAR}^{A},Descriptor_{Extension}^{A}\right)$9:  $Points^{A}\leftarrow Contour^{A}\left[Indices^{A}\right]$10:  $Contours^{B}\leftarrow$FindContours(B)11:  $Distance_{min}\leftarrow \infty$12:  **for** $Contour^{B}\leftarrow Contours_{i}^{B}$**do**              ▷ test each found contour13:    $Descriptor_{TAR}^{B},Indices^{B}\leftarrow$CalculateTAR$\left(Contour^{B}\right)$14:    $Descriptor_{Extension}^{B}\leftarrow$CalculateExtension$\left(Contour^{B},Indices^{B},B\right)$15:    $Points^{B}\leftarrow Contour^{B}\left[Indices^{B}\right]$16:    Distance,Pairing←Compare$\left(Descriptor^{B},Descriptor^{A}\right)$17:    **if** $Distance<Distance_{min}$ **then**18:      $Distance_{min}\leftarrow Distance$19:      $Pairing_{min}\leftarrow Pairing$20:      $Points_{min}\leftarrow Points^{B}$21:  **if** $Distance_{min}\neq \infty$ **then**22:    T←Align$\left(Points_{min},Points^{A}\left[Pairing_{min}\right]\right)$23:    **return** T                   ▷ return transform that align *B* to *A*

## 3. Results

We have tested our processing pipeline on several objects (precisely seven), having a few scans (precisely one time seven, four times five, and two times two) made under different conditions per each type. We selected one scan as a template and tested it with other ones. We tested the shape comparison mechanism per each type, having from one to ten contours per scan. We also tried to overcome our dataset’s small size by testing the shape comparison for all contours in the whole dataset, each to the other. In this way, we tried hundreds of comparisons, all successfully. The maximal distance between the same object shapes was smaller than the minimal distance between them and other objects’ contours for each tested item.

**Figure 8:**
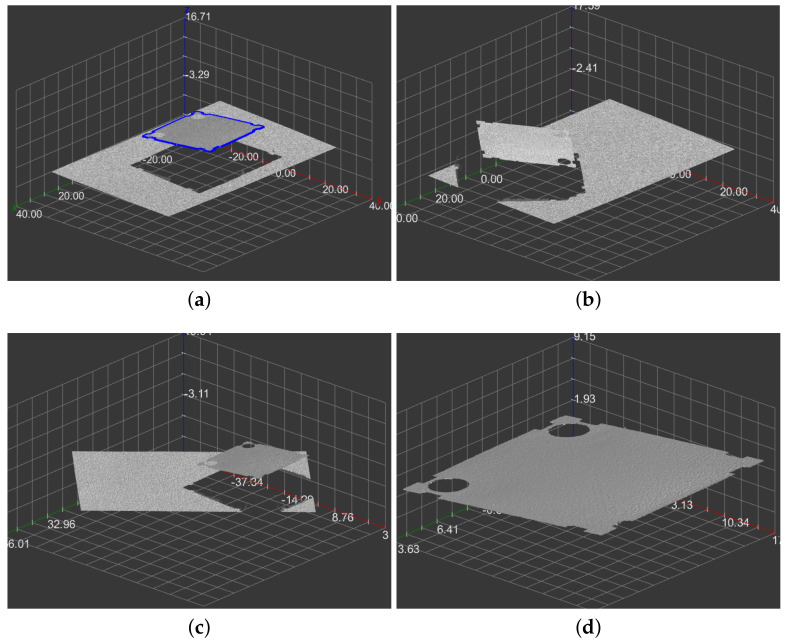
Results for a single input. (**a**) Contour selected for the template; (**b**) the input depth map; (**c**) the aligned depth map; (**d**) the recognized and aligned object.

**Figure 9:**
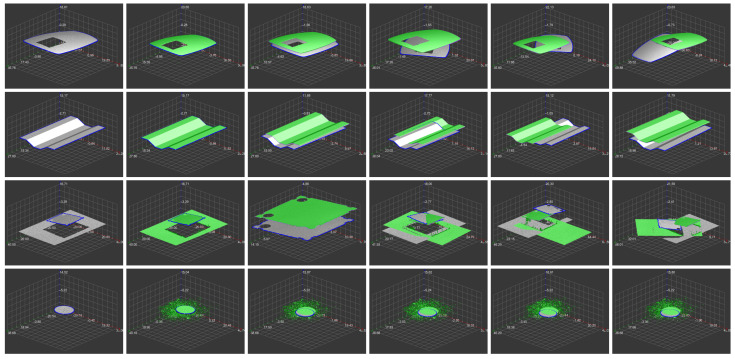
Here, we present results for several objects and their scans. Each line offers five scans of the same item, from which the first one we select as the template (the first column). The gray color represents measured values while the green one the aligned depth map (we align the whole depth map for better comparison; the recognized object could also be separated, of course).

We present the results for an example object in Figure 8 and more scans and objects in Figure 9. Table 1 presents quantitive results related to the shape comparison. We have tested our solution’s ability to recognize our object by the following distance of its shape to the samples’ shape of the same and different types. Hence, the first distance is smaller than the second ones, the shape comparison is correct. This fact is visible in the table—diagonal values are lower than others. Table 2 presents quantitive results related to the shape alignment quality. On average, we get an error of 0.35%. Even this deviation corresponds mainly to the discretization of data during the evaluation of the error and the fact that the actual data contours are not perfectly smooth. For comparison, the average error of alignment with itself is 0.20%. The processing time (mentioned in the same table) depends on the input data size, density, and contours. On a single-core CPU, we get e.g., 0.2 s, 0.3 s, or 0.7 s depending on the mentioned parameters. Moreover, the DSW algorithm, which is the most consuming part, can be implemented on GPU or employ parallelism on more CPU cores.

Following the quantitative evaluation in [27], we have tested our approach on the Kimia99 dataset [41,42]. The dataset contains various shapes that we have used as molds for the generation of depth maps. We randomly rotated and translated each depth map and tried to align it with the original one. Then we evaluated the error by the overlapping of the original and the aligned object. At first, we have checked that we get proper point-to-point pairing for 100% of samples. Then we evaluated the error for the alignment result. We get an error of 0.0045% (the depth maps are artificial; thus, we get a much lower error than on actual scans). If we replace our alignment algorithm with the classic approach, that is, with the single call of the Kabsch algorithm, we get a slightly higher error of 0.0086%. It means that for most of the samples, the Kabsh algorithm is sufficient, and there is no difference between its output and the output of our tuned version. Still, it sometimes exhibits significant error (see Figure 7), which is a big limitation for its industrial use. Therefore our tuned solution is much more applicable in practice.

## 4. Discussion

We live in an era of machine learning [43]. However, looking for a processing pipeline for a particular problem solicited from the industry, we often lack a larger dataset. Also, in our case, the requirement was to operate with a single example. Such conditions give a chance for the further development of classic methods. We had to rely on the correctness of our algorithms instead of statistical validation.

We had a problem clearly defined in the beginning, and we have been looking for technology for which it is tractable. We find that shape-based methods are most applicable and, among them, we found TAR as the best solution. The usage of existing methods appeared to be able to solve simple cases. Then we have to tackle problems that occurred in advanced cases.

We consider that the objects are products, and the aim of their alignment is mainly their quality inspection. We appreciate the preservation of shape during alignment, since, just in this case, we can compare the aligned object with the prototype looking for defects. It is an opposite requirement to that of a lot of applications that require accommodation of the compared contours. Of course, our approach allows the recognition of moderate deviations from the template contour. However, this is given just by a non-zero limit in the distance between TAR descriptors of the compared shapes.

We present our contribution to the current state-of-the-art in Table 3. We have selected several approaches with a similar mission. All of them are based on the shape comparison using various shape descriptors. Two of them employs TAR as we do. We hired comparison features of individual approaches relative to object recognition and alignment—the number of supported objects and their types, whether they can overlap, whether the supported shapes are simpler or complicated, the danger of the phantom detections. Of course, we also focused on the approaches’ ability to solve the problem with asymmetric aspects of the symmetric objects. Finally, we followed the precision of alignment. Our solution positively supports most of these features. Just regarding the overlapping, we are limited. Our items can overlap, but their contours must not. That is good enough for the concerned application purposes. However, we cannot process two pieces that overlap as the CSC can. On the other hand, using CSC, there is a significant danger of phantom detections. Thus our approach is more convenient for industrial applications.

A fundamental boundary of our approach is that the scanned objects must not overlap by their contours. One item can stand on top of another, but not at its edge. From limitations that we have not expected but found, we mention the quality of scans. In one case, we failed to find the proper contour. But indeed, the scanned data’s shape differed from reality. In practice, we had to consider that the laser profile sensor can fail in this way. Another limitation is the setup of data binarization. It is possible to volume for a particular object type, but any universal configuration cannot grant that the binarization is always successful. If we succeed in having the object contour in the binarized data, the rest of the process is thriving. A limitation also resides in the speed of processing. The cyclic DSW algorithm is quite time consumable and confines the number of the points that sample the contour. A reasonable number of sampling points is 200. It is also sufficient for complicated shapes, but it is a limitation.

Further development of our solution is possible mainly through the inclusion of other methods for contour finding. That is a part of our approach that could be improved by methods of deep learning. We could even consider an automated generation of a dataset using a generator of various solids and their processing by contour finding we have introduced in this paper.

An improvement is also possible in binarizing the object’s inner area for extension of the TAR descriptor. We rely upon that holes in the scanned item will contain some non-measured values. This assumption usually holds for holes. But the description of the inner area could be more accurate if we can also include bulges and cavities.

On the other hand, further improvement of the alignment algorithm is not very probable.

## 5. Conclusions

We have designed, implemented, tested and evaluated a processing pipeline for object recognition and alignment. We have combined several existing methods, contributed by overcoming their limitations, and created an integrated system with applicable quality for industrial purposes and potential for competitive products. We resolved all issues successfully, and our industrial partners found our results to be plausible. We summarize our contribution in three points:

- We have improved the TAR method by extending the TAR descriptor and making it operational for objects with symmetric shapes and other asymmetric aspects.
- We have designed an algorithm for contours alignment (preserving their shape), which utilizes the Kabsch algorithm and provides better results.
- We have integrated several methods into an operational processing pipeline.

Our processing pipeline is a module dedicated to usage within integrated inspection systems. Thanks to the module, such systems can perform quality tests on the aligned object straightforwardly, while it would be difficult to achieve the same with the measured data.

Our solution operates in real-time, that is, time consumption enables us to process each product on the production line. Our solution is also suitable for edge devices; we could even embed it into the laser profile sensor. Such intelligent sensors can directly send the quality test results to the production control system, simplifying inspection implementation.

We implemented our solution using the OpenCV Library [44]. We have used programming languages C++ and Python. We share selected codes of this project at GitHub (https://github.com/andylucny/ObjectAlignmentViaTAR).

## Figures and Tables

**Table 1:** The average shape distances for selected types of samples.

	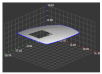	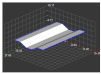	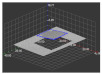	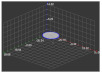
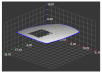	11.95	21.44	45.98	85.41
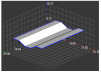	21.24	6.17	34.96	99.99
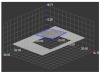	45.84	35.15	14.16	105.70
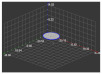	85.46	100.10	105.72	23.51

**Table 2:** The average result precision and time consumption of the whole processing pipeline for selected types of samples

	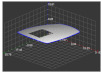	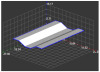	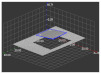	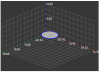
precision	0.35%	0.12%	0.49%	0.42%
time consumption	0.21s	0.21s	0.30s	0.75s

**Table 3:** Comparison of comparable approaches, concerning the current state-of-the-art. We have qualitatively compared several methods based on the TAR and other shape descriptors. We have considered several features listed in the left column and evaluated their presence or quality of the individual approaches.

Method:	Hough [18]	HSD [26]	CSC [33]	SIFT [13]	TAR [24]	TAR [25]	Our Approach
Number of objects:	one	many	many	one	one	one	many
Types of objects:	one	any	any	any	one	one	any
Overlapping objects:	N/A	no	yes	N/A	N/A	N/A	partially
Symmetric shape objects with asymmetric aspects handled correctly:	N/A	yes	yes	no	N/A	N/A	yes
Complicated shapes supported:	no	no	yes	yes	yes	yes	yes
Phantom detections’ danger:	none	low	high	low	none	none	none
Alignment of the recognized object:	yes	no	no	no	yes rough	yes rough	yes precise

## Data Availability

The data presented in this study are available at https://github.com/andylucny/ObjectAlignmentViaTAR. We have also employed the publicly available dataset KIMIA99 that can be found at https://github.com/mmssouza/kimia99.
